# Patient-level predictors of detection of depressive symptoms, referral, and uptake of depression counseling among chronic care patients in KwaZulu-Natal, South Africa

**DOI:** 10.1017/gmh.2020.11

**Published:** 2020-07-21

**Authors:** Christopher G. Kemp, Ntokozo Mntambo, Max Bachmann, Arvin Bhana, Deepa Rao, Merridy Grant, James P. Hughes, Jane M. Simoni, Bryan J. Weiner, Sithabisile Gugulethu Gigaba, Zamasomi Prudence Busisiwe Luvuno, Inge Petersen

**Affiliations:** 1 Department of Global Health, University of Washington, Seattle, WA, USA; 2 Centre for Rural Health, School of Applied Human Sciences, University of KwaZulu-Natal, Durban, South Africa; 3 Norwich Medical School, University of East Anglia, Norwich, Norfolk, UK; 4 SA Medical Research Council, Health Systems Research Unit, Durban, South Africa; 5 Department of Psychiatry and Behavioral Medicine, University of Washington, Seattle, WA, USA; 6 Department of Biostatistics, University of Washington, Seattle, WA, USA; 7 Department of Psychology, University of Washington, Seattle, WA, USA

**Keywords:** Chronic disease, depression, HIV, integrated care, primary health care, South Africa

## Abstract

**Background:**

Integration of depression treatment into primary care could improve patient outcomes in low-resource settings. Losses along the depression care cascade limit integrated service effectiveness. This study identified patient-level factors that predicted detection of depressive symptoms by nurses, referral for depression treatment, and uptake of counseling, as part of integrated care in KwaZulu-Natal, South Africa.

**Methods:**

This was an analysis of baseline data from a prospective cohort. Participants were adult patients with at least moderate depressive symptoms at primary care facilities in Amajuba, KwaZulu-Natal, South Africa. Participants were screened for depressive symptoms prior to routine assessment by a nurse. Generalized linear mixed-effects models were used to estimate associations between patient characteristics and service delivery outcomes.

**Results:**

Data from 412 participants were analyzed. Nurses successfully detected depressive symptoms in 208 [50.5%, 95% confidence interval (CI) 38.9–62.0] participants; of these, they referred 76 (36.5%, 95% CI 20.3–56.5) for depression treatment; of these, 18 (23.7%, 95% CI 10.7–44.6) attended at least one session of depression counseling. Depressive symptom severity, alcohol use severity, and perceived stress were associated with detection. Similar factors did not drive referral or counseling uptake.

**Conclusions:**

Nurses detected patients with depressive symptoms at rates comparable to primary care providers in high-resource settings, though gaps in referral and uptake persist. Nurses were more likely to detect symptoms among patients in more severe mental distress. Implementation strategies for integrated mental health care in low-resource settings should target improved rates of detection, referral, and uptake.

## Introduction

An estimated 75% of South Africans experiencing mental disorder never receive any kind of mental health treatment (Seedat *et al*., 2009). Common mental disorders (CMDs) like depression, anxiety, and alcohol abuse frequently coexist with the growing syndemics of HIV and other chronic diseases (Myer *et al*., 2008; Pappin *et al*., 2012; Bhana *et al*., 2015) and limit the effectiveness of therapies for these physical diseases by reducing medication adherence and retention in care (Peltzer *et al*., 2010; Nakimuli-Mpungu *et al*., 2012). South Africans who do seek mental health services are over three times more likely to do so from a general medical provider than from a mental health specialist (Seedat *et al*., 2009). Integration of evidence-based mental health care into routine chronic disease services at the primary health care (PHC) level has been advanced by the South African National Mental Health Policy Framework and Action Plan (2013–2020) as an important strategy to reduce the treatment gap and improve associated mental and physical health outcomes (Collins *et al*., 2011; National Department of Health, 2014).

Evidence from high-income countries suggests that collaborative models for the integration of mental health care into the PHC platform can be cost-effective for patients with co-morbid chronic illness (Woltmann *et al*., 2012). Integrated care models have also demonstrated promising effectiveness in South Africa (Petersen *et al*., 2019). PHC-based professional nurses – clinical nurse practitioners (CNPs) – are at the core of the South African model for integrated care: they are responsible for detecting patients with co-morbid CMDs during routine care for chronic diseases like HIV and hypertension, providing brief psycho-education, referring patients into appropriate mental health treatment, and managing their ongoing care (Fairall *et al*., 2018; Petersen *et al*., 2018). Implementation strategies for this model include training CNPs in the use of a national standard clinical decision support tool, outreach and change management workshops with clinic staff, and the addition of in-facility counseling by trained lay counselors. However, early evidence from this model suggested that, once trained, CNPs were able to positively detect only 16–27% of patients with depression (Petersen *et al*., 2019). Such losses along the depression care cascade threaten the overall effectiveness of integrated care in this context. Both CNPs and patients face unique, multi-level barriers to delivery and receipt of services for depression and other CMDs (Anthony *et al*., 2010); identification of these barriers will enable targeted refinements to the integrated care model and its implementation strategy (Jacobs *et al*., 2015).

Patient-level predictors of detection, referral, and treatment uptake are particularly relevant given that integrated care models require providers to holistically meet the diverse needs of patients with multiple co-morbidities (Watt *et al*., 2017). It is therefore important to understand whether variation in patient sociodemographic characteristics, clinical presentation, and medical history predicts whether patients successfully receive treatment. Our objective was to identify patient-level factors that predicted successful detection of depressive symptoms by CNPs, referral for depression-related treatment, and uptake of depression counseling, as part of integrated primary mental health care in KwaZulu-Natal, South Africa.

## Methods

### Conceptual model

Our approach was informed by the idea of the depression treatment cascade; that is, among patients in primary care with co-morbid chronic disease and depression, a proportion will be detected as having probable depression by providers; a proportion of those will be referred into appropriate depression treatment; a proportion of those will take up treatment; and a proportion of those will experience remission (Pence *et al*., 2012). Patient losses are thus anticipated at each level of the cascade. By targeting steps with large losses – and modifiable factors that predicted those losses – overall rates of depression remission can be improved. This study investigates the first three steps: detection, referral, and treatment uptake.

### Integrated care model

An integrated, collaborative approach to the management of depression and other CMDs was developed and evaluated in South Africa with the support of the Programme for Improving Mental Healthcare (PRIME) consortium [40]. The history and design of this model have been described in detail elsewhere (Fairall *et al*., 2018; Petersen *et al*., 2018). It was piloted and trialed in North West Province, and scaled to Amajuba District in KwaZulu-Natal with the support of the President's Emergency Plan For AIDS Relief (PEPFAR). The model was built from South Africa's efforts to deliver the majority of care for chronic disease at a single PHC-level service point. It involved strengthening the capacity of CNPs to identify and manage patients with depression and other CMDs, and of sessional PHC physicians to treat CMDs; training PHC-based lay behavioral health counselors to provide up to eight sessions of individual- or group-based evidence-based counseling; and reinforcing necessary referral pathways, monitoring systems, and supervisory structures. In Amajuba, lay HIV counselors at each facility were trained as behavioral health counselors. Implementation of the model in Amajuba was supported by a cross-cutting, health systems strengthening approach. Continuous quality improvement methods were used to overcome early bottlenecks and strengthen the system's capacity to provide mental health services alongside services for other chronic diseases.

CNPs received supplemental training in clinical communication skills and in the mental health components of a national standard clinical decision support tool (Adult Primary Care, or APC) (Fairall *et al*., 2015). Training was provided through a train-the-trainer approach: nurse master trainers at each PHC were trained to deliver four sessions of supplemental material to their peers. The APC tool includes algorithms and checklists developed to support nurse-led identification and management of all common chronic diseases, including depression and other CMDs. CNPs use the steps outlined in the APC tool to screen chronic care patients for depression and other CMDs, make an assessment based on clinical presentation and patient medical history, provide brief psycho-education, write appropriate referrals, and manage ongoing care. They may refer directly to the APC flowcharts during patient consultations, or they may review the flowcharts before and after patient consultations. Based on their assessment, CNPs can refer patients to the PHC-based behavioral counselor. Depending on case severity, they can also refer patients for diagnostic confirmation and/or treatment by a sessional physician or district hospital-based psychologist.

### Data source

We analyzed baseline data from the prospective cohort of the implementation study of the scale-up of the integrated care model described above (Petersen *et al*., 2019). This cohort was part of the Southern African Research Consortium for Mental health INTegration (S-MhINT). From April to October 2018, patients attending chronic disease services at 10 different PHC facilities in Amajuba District, KwaZulu-Natal, South Africa, were invited to participate in the cohort study. These facilities had been implementing the integrated collaborative mental health model since late 2017. Amajuba District's public health system covers a mostly peri-urban population, was one of KwaZulu-Natal's three provincial pilot sites for South Africa's National Health Insurance program, and was noted as high-performing by the country's Ideal Clinic Monitoring System (Department of Health, 2019).

Trained fieldworkers administered a brief screening tool, including assessments of depressive symptoms (PHQ-9) and alcohol use (AUDIT), to interested patients. Both have been validated for use in South African populations (Myer *et al*., 2008; Bhana *et al*., 2015). Patients were eligible for enrollment in the cohort if they were at least 18 years old; had the time and ability to complete the baseline interview; and scored ⩾9 on the PHQ-9 and/or ⩾8 on the AUDIT. Patients were excluded from enrollment if they were unable to provide informed consent (e.g. in case of severe intellectual disability, acute medical issue, or lack of private space) or were planning to leave the study area within 12 months. Most cohort participants – and all participants included in this analysis – were recruited from the chronic care waiting room, prior to routine assessment and consultation with the CNP. In an effort to increase the cohort sample size of patients who had received a referral for depression treatment, the fieldworkers subsequently recruited some participants post-referral. Facility-based behavioral counselors notified fieldworkers when they received new patient referrals to that fieldworkers could approach the referred patients directly, prior to their scheduled first counseling session. Participants were only eligible for this current analysis if they were enrolled prior to routine assessment by the CNP (i.e. their enrollment was not conditional on detection or referral), and if they scored ⩾9 on the PHQ-9. Participants recruited post-referral were excluded.

During screening, if patients responded positively to the suicidal ideation question of the PHQ-9, fieldworkers repeated the question to confirm the response. Patients reporting suicidal thoughts in the past two weeks were given psychoeducational material and contact details for accessing help, and those reporting suicidal thoughts on more than seven days in the past two weeks were referred directly to the CNP for assessment and onward referral to the sessional physician or psychologist if necessary.

Following written informed consent, participants completed a baseline questionnaire in their choice of English or isiZulu. Questionnaire data were collected using tablets. With the exception of suicidal ideation, as noted above, screening results, cohort enrollment, and questionnaire data were not reported to CNPs; CNPs assessed and cared for patients as usual, blinded to the screening results. Fieldworkers then tracked the mental health-related service delivery outcomes of enrolled patients, including CNP detection of depressive symptoms and referral for depression-related treatment, using CNP-completed checklists and patient records. All study procedures were approved by the Biomedical Research Ethics Committee of the University of KwaZulu-Natal (BF190/17) and by the Department of Health of the Province of KwaZulu-Natal.

### Outcomes

We assessed three outcomes of interest. The first was detection of depressive symptoms by the nurse, defined as the PHC-based CNP noting the depressive symptoms in the patient record or indicating on the checklist that the patient had a probable case of depression. The second was referral for depression-related treatment, defined as the CNP completing a referral form for counseling by the PHC-based behavioral counselor. Referrals may also be made for diagnostic confirmation and/or treatment by a PHC-based physician or district hospital-based psychologist. The second outcome was conditional on the first, as a referral could not be made without first detecting the depressive symptoms. The third was uptake of depression-related counseling from a PHC-based behavioral counselor. For the purpose of this analysis, uptake was defined as attendance of at least one session; this served to maximize sample size across the outcome strata, given low uptake of subsequent sessions. The third outcome was conditional on the first and second outcomes, as counseling uptake could not occur without both detection and referral.

### Predictors

We examined predictors related to patient socio-demographic characteristics, healthcare use, prior chronic disease diagnoses, assessment of the quality of chronic illness care, perceived stress, social support, general disability, and clinical characteristics. Socio-demographic characteristics were age, sex, race, educational attainment, employment status, employment and social grant-related income, and self-reported household food insecurity. Healthcare use was measured by self-report of the number of PHC visits and number of hospitalizations in the previous 3 months. Prior diagnoses were measured by self-report and included depression, HIV, asthma, chronic obstructive pulmonary disease, hypertension, heart disease, stroke, tuberculosis, diabetes, high cholesterol, arthritis, epilepsy, and other chronic and mental disorders. Assessment of prior chronic illness care was measured using the Patient Assessment of Chronic Illness Care (PACIC), which evaluates the quality of care and alignment with the chronic care model (Glasgow *et al*., 2005). Perceived stress was measured using the perceived stress scale (PSS), which assesses the extent to which situations in a person's life are appraised as stressful (Cohen *et al*., 1983). Social support was measured using the Oslo social support scale (Dalgard *et al*., 2006); this three-item instrument asks about numbers of close confidants, sense of concern from other people, and relationships with neighbors, with a focus on the availability of practical help. General disability was measured with the 12-item interviewer-administered version of the WHO Disability Assessment Schedule (WHODAS), which is a generic assessment instrument for disability for diseases including mental disorders (Garin *et al*., 2010). Clinical characteristics included depressive symptom and alcohol use severity. Depressive symptoms were measured with the PHQ-9, which is aligned with the Diagnostic and Statistical Manual (DSM-IV-TR) diagnostic criteria for major depressive disorder (Kroenke *et al*., 2010). Scores between 9 and 14 indicated moderate depression, between 15 and 19 indicated moderately severe depression, and between 20 and 27 indicated severe depression. Alcohol use was measured with the AUDIT (Saunders *et al*., 1993). All measures have been previously used and found to be valid in South Africa ( Myer *et al*., 2008; Garin *et al*., 2010; Vythilingum *et al*., 2012; Bhana *et al*., 2015).

### Statistical analysis

Descriptive analyses, including univariate logistic regression models adjusting for clustering at the health facility level, were conducted to summarize and compare participant characteristics stratified by service delivery outcome: not detected *v*. detected, not referred *v*. referred, and no treatment uptake *v*. any treatment uptake. We then used generalized linear mixed-effects models to estimate associations between the predictors of interest and the nested service delivery outcomes. All three models used the binomial family and logit link and included random facility-specific intercepts to adjust for clustering. As a sensitivity test for type II error, we included parsimonious models with only covariates with adjusted *p* values < 0.1. Finally, we used the model estimates to calculate and plot the predicted probabilities of detection, referral, and uptake over the range of PHQ-9 scores observed at screening, with all other covariates at their means (King *et al*., 2000).

All analyses were performed in Stata and R (R Core Team, 2017; StataCorp, 2015). Statistical significance was defined at *α* = 0.05.

## Results

**Fig. 1.**
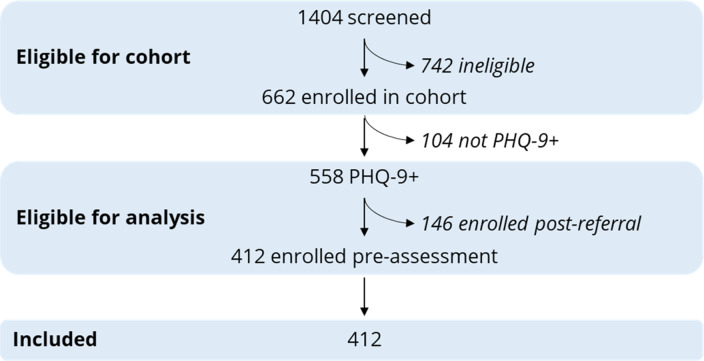
Cohort recruitment flow diagram.

**Fig. 2.**
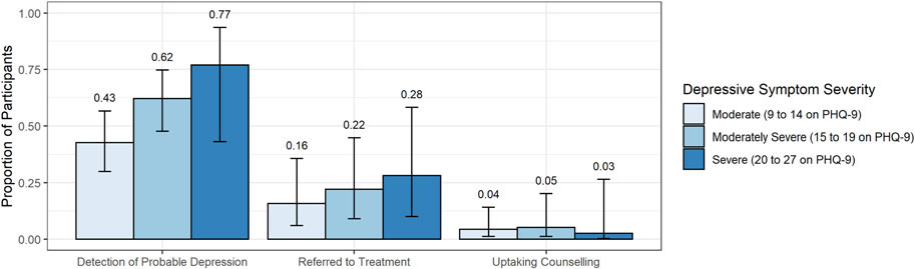
Depression care cascade stratified by depressive symptom severity. Note: Error bars represent 95% confidence intervals.

We screened 1404 patients for study eligibility; 662 patients were eligible for enrollment and recruited into the cohort (Fig. 1). Five hundred and fifty-eight participants had PHQ-9 scores ⩾ 9, of whom 412 were enrolled prior to CNP assessment and were eligible for this analysis. Overall, CNPs detected depressive symptoms in 208 [50.5%, cluster-adjusted 95% confidence interval (CI) 38.9–62.0] participants; of these, they referred 76 (36.5%, 95% CI 20.3–56.5) for depression treatment; of these, 18 (23.7%, 95% CI 10.7–44.6) received at least one session of depression counseling. Overall, 18.4% (95% CI 9.0–34.2) of participants were referred for depression treatment, and 4.4% (95% CI 1.6–11.5) of participants received at least one session of depression counseling. On average, participants receiving any treatment attended 1.7 sessions (range 1–8); one participant completed all eight sessions. Participants with moderately severe (62.1%, 95% CI 47.6–74.7) or severe depressive symptoms (76.9%, 95% CI 43.2–93.6) were more likely to be detected compared to participants with moderate depressive symptoms (42.8%, 95% CI 30.0–56.6) (Fig. 2, *p* = 0.013). Among detected participants, participants with moderate (37.0%, 95% CI 16.8–63.0), moderately severe (35.6%, 95% CI 15.8–61.9), and severe depressive symptoms (36.7%, 95% CI 14.6–66.2) were similarly likely to be referred for treatment (*p* = 0.99). Among referred participants, participants with moderate (27.3%, 95% CI 9.5–57.3), moderately severe (23.8%, 95% CI 10.2–46.2), and severe depressive symptoms (9.1%, 95% CI 0.5–67.0) were similarly likely to uptake treatment (*p* = 0.52).

The mean participant age was 44.3 years [standard deviation (s.d.) 13.2] (Table 1). The majority were female (337, 81.8%), almost all were Black (410, 99.5%), and few (88, 21.4%) had matriculated from high school. Overall, the majority (278, 67.5%) had moderate depressive symptoms, and most (332, 80.6%) had low-risk alcohol use. Participants taking up treatment were more likely to have low household income [⩽2000 South African Rand (ZAR) per month] compared to participants not taking up treatment (83.3% *v*. 70.7%, *p* = 0.020), and more likely to report household food insecurity (66.7% *v*. 46.6%, *p* = 0.036). Few (21, 5.1%) participants had been hospitalized in the previous 3 months. The majority had an HIV diagnosis prior to study enrollment (318, 77.6%); detected participants were slightly more likely to have an HIV diagnosis (80.3% *v*. 74.8%, *p* = 0.040) and to have reported a prior diagnosis of depression compared to undetected participants (8.2% *v*. 3.0%, *p* = 0.020). Both diagnosed and referred participants were less likely than un-detected and un-referred participants to have reported a prior diagnosis for a chronic disease other than HIV, hypertension, or diabetes (37.2% *v*. 28.1%, *p* = 0.019; 26.7% *v*. 43.2%, *p* *=* 0.005, respectively). Over one-third (161, 39.2%) of all participants reported prior diagnoses of two or more chronic diseases. Almost none were currently taking anti-depressant medication (3, 0.7%). Participants perceived the quality of their chronic care to be low overall (mean PACIC 0.44, s.d. 0.48). Detected participants were more likely than undetected participants to have reported high stress (30.0% *v*. 15.8%, *p* = 0.005) and to have reported higher disability (WHODAS score of 0.28 *v*. 0.22, *p* = 0.014). They were also more likely to have severe depressive symptoms (14.4% *v*. 4.4%, *p* = 0.013), to report any suicidal thoughts (48.1% *v*. 28.4%, *p* = 0.03), and to have high-risk and dependent alcohol use (11.5% *v*. 2.5%, *p* = 0.002). Participants receiving a referral for depression treatment were less likely than those detected but not referred to report high social support (9.3% *v*. 16.8%, *p* < 0.001).

**Table 1.** Participant characteristics stratified by detection, referral, and uptake (*n* = 412)

	Detection			Referral (given detection)			Uptake (given referral)			Total
Factor	Not detected	Detected	*p*^a^	Not referred	Referred	*p*^a^	No uptake	Any uptake	*p*	
*N*	204	208		132	76		58	18		412
Age, mean (s.d.)	44.6 (13.6)	44.0 (12.8)	0.69	44.5 (12.8)	43.2 (13.0)	0.39	43.8 (14.0)	41.5 (8.8)	0.13	44.3 (13.2)
Female	171 (83.8%)	166 (79.8%)	0.23	106 (80.3%)	60 (78.9%)	0.82	45 (77.6%)	15 (83.3%)	0.64	337 (81.8%)
Black	202 (99.0%)	208 (100%)		132 (100%)	76 (100%)		58 (100%)	18 (100%)		410 (99.5%)
Matriculated	41 (20.1%)	47 (22.6%)	0.38	31 (23.5%)	16 (21.1%)	0.67	13 (22.4%)	3 (16.7%)	0.60	88 (21.4%)
Employed	60 (29.4%)	52 (25.0%)	0.37	35 (26.5%)	17 (22.4%)	0.32	12 (20.7%)	5 (27.8%)	0.31	112 (27.2%)
Low income (2000 ZAR/month)	134 (65.7%)	151 (72.6%)	0.051	95 (72.0%)	56 (73.7%)	0.69	41 (70.7%)	15 (83.3%)	**0.020**	285 (69.2%)
Household food insecurity	84 (41.4%)	98 (47.1%)	0.24	59 (44.7%)	39 (51.3%)	0.39	27 (46.6%)	12 (66.7%)	**0.036**	182 (44.3%)
Healthcare use in previous 3 months										
Other PHC visit	131 (64.2%)	127 (61.1%)	0.69	83 (62.9%)	44 (57.9%)	0.69	31 (53.4%)	13 (72.2%)	0.38	258 (62.6%)
Hospitalized	12 (5.9%)	9 (4.3%)	0.181	5 (3.8%)	4 (5.3%)	0.64	2 (3.4%)	2 (11.1%)	0.20	21 (5.1%)
Prior diagnoses										
HIV	151 (74.8%)	167 (80.3%)	**0.040**	104 (78.8%)	63 (82.9%)	0.47	46 (79.3%)	17 (94.4%)	0.10	318 (77.6%)
Depression	6 (3.0%)	17 (8.2%)	**0.020**	8 (6.1%)	9 (11.8%)	0.36	9 (15.5%)	0		23 (5.6%)
Hypertension	58 (28.7%)	52 (25.0%)	0.38	39 (29.5%)	13 (17.1%)	**0.026**	10 (17.2%)	3 (16.7%)	0.94	110 (26.8%)
Diabetes	16 (7.9%)	19 (9.1%)	0.49	14 (10.6%)	5 (6.6%)	0.39	3 (5.2%)	2 (11.1%)	0.37	35 (8.5%)
Other chronic disease	57 (28.1%)	77 (37.2%)	**0.019**	57 (43.2%)	20 (26.7%)	**0.005**	16 (28.1%)	4 (22.2%)	0.66	134 (32.7%)
Number of prior chronic disease diagnoses			**0.039**			**0.049**			0.46	
0	2 (1.0%)	1 (0.5%)		1 (0.8%)	0		0	0		3 (0.7%)
1	131 (64.5%)	116 (55.8%)		68 (51.5%)	48 (63.2%)		35 (60.3%)	13 (72.2%)		247 (60.1%)
⩾2	70 (34.5%)	91 (43.8%)		63 (47.7%)	28 (36.8%)		23 (39.7%)	5 (27.8%)		161 (39.2%)
On depression medication	2 (1.0%)	1 (0.5%)	0.54	0	1 (1.3%)		1 (1.7%)	0		3 (0.7%)
PACIC, range 0–3, mean (s.d.)	0.45 (0.47)	0.44 (0.49)	0.97	0.41 (0.46)	0.50 (0.54)	0.46	0.47 (0.51)	0.60 (0.64)	0.09	0.44 (0.48)
PSS			**0.005**			0.72			0.33	
Low stress	25 (12.4%)	4 (1.9%)		4 (3.1%)	0		0	0		29 (7.1%)
Moderate stress	145 (71.8%)	141 (68.1%)		87 (66.4%)	54 (71.1%)		43 (74.1%)	11 (61.1%)		286 (69.9%)
High stress	32 (15.8%)	62 (30.0%)		40 (30.5%)	22 (28.9%)		15 (25.9%)	7 (38.9%)		94 (23.0%)
Oslo social support			0.13			**<0.001**			0.13	
Poor support	87 (43.1%)	102 (49.5%)		57 (43.5%)	45 (60.0%)		30 (52.6%)	15 (83.3%)		189 (46.3%)
Moderate support	74 (36.6%)	75 (36.4%)		52 (39.7%)	23 (30.7%)		21 (36.8%)	2 (11.1%)		149 (36.5%)
Strong support	41 (20.3%)	29 (14.1%)		22 (16.8%)	7 (9.3%)		6 (10.5%)	1 (5.6%)		70 (17.2%)
WHODAS, range 0–1, mean (s.d.)	0.22 (0.18)	0.28 (0.19)	**0.014**	0.27 (0.19)	0.28 (0.20)	0.83	0.28 (0.21)	0.31 (0.15)	0.45	0.25 (0.19)
PHQ-9			**0.013**			0.99			0.52	
Moderate depression	159 (77.9%)	119 (57.2%)		75 (56.8%)	44 (57.9%)		32 (55.2%)	12 (66.7%)		278 (67.5%)
Moderately severe depression	36 (17.6%)	59 (28.4%)		38 (28.8%)	21 (27.6%)		16 (27.6%)	5 (27.8%)		95 (23.1%)
Severe depression	9 (4.4%)	30 (14.4%)		19 (14.4%)	11 (14.5%)		10 (17.2%)	1 (5.6%)		39 (9.5%)
Any suicidal thoughts	58 (28.4%)	100 (48.1%)	**0.03**	70 (53.0%)	30 (39.5%)	0.053	21 (36.2%)	9 (50.0%)	0.17	158 (38.3%)
AUDIT			**0.002**			0.21			0.47	
Low risk	170 (83.3%)	162 (77.9%)		99 (75.0%)	63 (82.9%)		49 (84.5%)	14 (77.8%)		332 (80.6%)
Risky or hazardous	24 (11.8%)	15 (7.2%)		10 (7.6%)	5 (6.6%)		4 (6.9%)	1 (5.6%)		39 (9.5%)
High-risk or harmful	5 (2.5%)	7 (3.4%)		6 (4.5%)	1 (1.3%)		1 (1.7%)	0		12 (2.9%)
High-risk and dependent	5 (2.5%)	24 (11.5%)		17 (12.9%)	7 (9.2%)		4 (6.9%)	3 (16.7%)		29 (7.0%)

s.d., standard deviation; ZAR, South African Rand; PHC, primary healthcare; PACIC, Patient Assessment of Care for Chronic Conditions scale; PSS, perceived stress scale; WHODAS, WHO Disability Assessment Schedule; PHQ-9, Patient Health Questionnaire-9; AUDIT, Alcohol Use Disorders Identification Test.

a *p* values adjusted for clustering by health facility. Bold indicates *p* < 0.05.

Regression models found that depressive symptom severity, suicidal thoughts, and perceived stress were all independently associated with detection by the CNP (Table 2). Specifically, each additional point in PHQ-9 score was associated with a 12% increase in odds of detection [adjusted odds ratio (aOR) 1.12, 95% CI 1.04–1.20], reporting any suicidal thoughts was associated with a 78% increase in odds of detection (aOR 1.78, 95% CI 1.02–3.11), and each additional point in PSS score was associated with an 11% increase in odds of detection (aOR 1.11, 95% CI 1.06–1.17). No other predictors were independently, statistically significantly associated with detection. In contrast, prior diagnosis of a chronic disease other than HIV or depression was the only predictor significantly associated with referral for depression treatment, conditional on detection: prior diagnosis of another chronic disease was associated with 57% reduced odds of referral (aOR 0.43, 95% CI 0.18–0.99). No other predictors were independently, statistically significantly associated with referral. Finally, social support was the only predictor significantly associated with uptake of depression counseling, conditional on referral: each point of additional Oslo score was associated with a 39% reduction in odds of any treatment uptake (aOR 0.61, 95% CI 0.40–0.92). Sensitivity analyses using parsimonious models supported these findings (online Supplemental Table S1).

**Table 2.** Generalized linear mixed-effects model estimates of predictors of detection, referral, and uptake

	Detected (*v*. not detected)			Referred (*v*. not referred)			Any uptake (*v*. no uptake)		
Predictors	aOR	95% CI	*p*	aOR	95% CI	*p*	aOR	95% CI	*p*
(Intercept)	0.01	0.00–0.08	**<0.001**	0.06	0.00–2.36	0.132	0	0.00–0.67	**0.04**
Age, years	1.01	0.99–1.03	0.396	1	0.97–1.03	0.99	1.05	0.96–1.16	0.268
Female	0.76	0.40–1.45	0.407	0.64	0.25–1.67	0.366	6.9	0.46–104.42	0.164
Matriculated	1.4	0.75–2.59	0.29	0.94	0.37–2.38	0.903	5.77	0.43–78.25	0.188
Employed	1.17	0.58–2.36	0.667	0.94	0.33–2.70	0.909	8.77	0.61–126.10	0.11
Income >2000 ZAR/month	0.97	0.50–1.89	0.926	0.63	0.23–1.69	0.356	4.3	0.33–56.75	0.268
Household food insecurity	1.19	0.72–1.98	0.498	1.72	0.80–3.70	0.167	4.33	0.43–43.79	0.214
Healthcare use in last 3 months									
Other PHC use	1.15	0.65–2.03	0.64	0.87	0.38–2.01	0.744	2.8	0.48–16.29	0.252
Hospitalized	0.54	0.18–1.61	0.271	0.8	0.16–4.11	0.788	31.15	0.44–2204.28	0.114
Prior diagnoses									
HIV	1.54	0.74–3.20	0.252	0.99	0.34–2.88	0.99	14.84	0.63–347.73	0.094
Depression	1.29	0.41–4.00	0.664	3.09	0.78–12.25	0.108	0	0.00–Inf	0.993
Other chronic disease	1.35	0.75–2.46	0.317	0.43	0.18–0.99	**0.047**	1.04	0.11–9.57	0.97
PACIC score	1.15	0.63–2.10	0.644	1.02	0.42–2.48	0.965	2.6	0.46–14.68	0.28
PSS score	1.11	1.06–1.17	**<0.001**	1.07	0.98–1.17	0.147	1.15	0.96–1.39	0.132
Oslo score	0.98	0.88–1.09	0.709	1.07	0.91–1.27	0.392	0.61	0.40–0.92	**0.017**
WHODAS score	0.6	0.13–2.77	0.514	1.65	0.17–15.74	0.663	11.54	0.02–5614.99	0.439
PHQ-9 score	1.12	1.04–1.20	**0.004**	1.04	0.94–1.16	0.402	0.78	0.59–1.03	0.076
Suicidal thoughts	1.78	1.02–3.11	**0.041**	0.57	0.25–1.33	0.195	1.22	0.14–10.92	0.858
AUDIT score	1.04	1.00–1.08	0.063	1.01	0.96–1.05	0.807	1.08	0.98–1.21	0.135
Random effects									
*σ*^2^	3.29			3.29			3.29		
*τ*_00_	1.11_facility_			2.31_facility_			2.33_facility_		
ICC	0.25			0.41			0.41		
Observations	402			203			75		

^a^OR, adjusted odds ratio; CI, confidence interval; ZAR, South African Rand; PHQ-9, Patient Health Questionnaire-9; AUDIT, Alcohol Use Disorders Identification Test; PHC, primary healthcare; PSS, perceived stress scale; WHODAS, WHO Disability Assessment Schedule; ICC, intraclass correlation. Bold indicates *p* < 0.05.

**Fig. 3.**
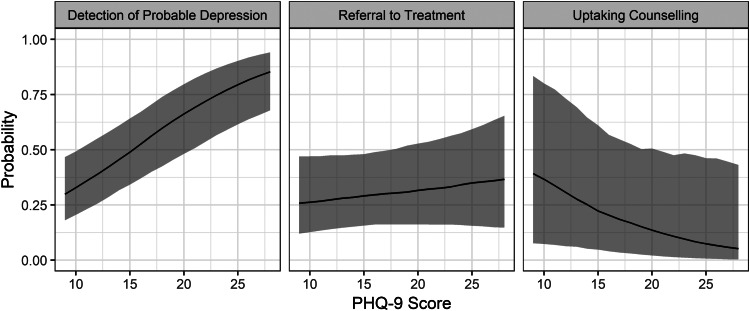
Predicted conditional probabilities of depression detection, treatment referral, and counseling uptake by depressive symptom severity. Note: Shaded areas represent 95% confidence intervals.

The predicted probability of depressive symptom detection rose from 29.8% (95% CI 18.9–43.4) at the lowest level of observed depressive symptoms to 93.8% (95% CI 83.0–97.8) at the highest level, holding all other covariates at their means (Fig. 3). In contrast, the predicted probability of treatment referral rose from 24.4% (95% CI 10.9–46.1) at the lowest level of observed depressive symptoms to 42.2% (95% CI 14.4–75.9) at the highest level, holding all other covariates at their means. Finally, the predicted probability of counseling uptake fell from 31.4% (95% CI 6.4–78.2) at the lowest level of observed depressive symptoms to 3.8% (95% CI 0.1–54.2) at the highest level, holding all other covariates at their means.

## Discussion

This study is among the first to characterize disparities in depression detection, referral, and counseling uptake, across patient sociodemographic and clinical characteristics, in an low- or middle-income country setting. Patients in the sample were mostly Black, low-income women living with HIV and experiencing moderate depressive symptoms. Many had two or more chronic disease diagnoses, though few had a prior diagnosis of depression. CNPs successfully detected depressive symptoms in nearly half of the sample; among those they detected, they referred just over one-third into treatment; among those with a referral, only one-quarter received any depression counseling. CNPs were more likely to detect depressive symptoms in patients with greater depressive symptom severity, alcohol use severity, and perceived stress, independent of other patient-level clinical and socio-demographic characteristics. These same factors did not drive referral into treatment or counseling uptake, though lower-income patients and those with lower social support were somewhat more likely to receive referrals and uptake treatment.

These results reflect a substantially higher rate of successful nurse detection compared to the rate found by the PRIME evaluation of this model in North West province (Petersen *et al*., 2019); indeed, this rate of detection closely matches global findings suggesting that, on average, PHC-based providers correctly detect depressive symptoms about half of the time (Mitchell *et al*., 2009). The rate of referral seen in this study also closely matches rates of treatment initiation proximal to mood disorder onset seen in other high- and middle-income settings (Wang *et al*., 2005; Wang *et al*., 2007; Thornicroft *et al*., 2017). The clinical interventions and provider-level implementation strategies used to support the integrated care model were the same in this study and in the PRIME evaluation. Contextual differences between the two districts may explain the comparably high detection rate observed by this study: as noted above, the health system of Amajuba district is considered high-performing (Department of Health, 2019). Moreover, the use of systems-level continuous quality improvement methods to promote buy-in among program and operational managers was novel in Amajuba. Both differences suggest that detection rates are higher given a supportive, well-functioning health system and appropriate levels of organizational support.

Still, many patients were missed. Although studies in the USA have identified significant disparities across the depression treatment cascade by sex and race (Simpson *et al*., 2007; Bengtson *et al*., 2016), we found no evidence that sociodemographic characteristics were associated with the likelihood of detection or referral in this context. Instead, nurses in our study appeared to be more influenced by clinical characteristics rather than sociodemographic characteristics. For example, we found that alcohol use symptoms were positively associated with detection of depressive symptoms, in contrast with findings from other settings (DiPrete *et al*., 2019). Interestingly, perceived stress and depressive symptom severity were both independently associated with detection of depressive symptoms, suggesting that PHC-based CNPs may interpret signs of stress as indicators of depression. However, mental distress was not a key determinant of referral or uptake, and overall probabilities of referral and uptake were low even among patients with severe depressive symptoms and suicidal ideation. We hypothesize that contextual determinants of implementation success at the nurse-, facility-, system-, and community-levels may be inhibiting these outcomes. For example, rates of successful referral and treatment uptake likely relate to the competence of CNPs in the assessment of patient mental health, the time available to CNPs to complete referral forms, the competence of facility-based behavioral counselors, and their availability and accessibility to referred patients when needed. This suggests that there may be opportunities to strengthen or enhance the implementation of the integrated care model in this setting, and that future research will need to assess higher-level predictors of referral and uptake.

This study had several limitations. First, in the absence of diagnostic assessment by psychiatrists or equivalent professionals, we relied on screening by fieldworkers using the PHQ-9 as an indicator of depressive symptoms and probable depression. This approach may have led to misclassification of some patients; we cannot report the true number of patients meeting criteria for a depressive disorder, nor can we report the true number of patients needing specialty mental health treatment. However, given that the process of nurse-led identification of depressive symptoms described in the APC manual is intended to be comparable to screening, and not to professional diagnostic assessment, the comparisons made in this analysis are valid. Second, the relatively small numbers of detected and referred participants limited our power to identify statistically significant predictors of referral conditional on detection or uptake conditional on referral: only 51% of our overall sample were detected, and only 18% were referred for counseling, leaving sample sizes of 208 and 76, respectively. Nonetheless, the descriptive statistics presented above provide a basis for future inquiry into patient-level predictors of referral and treatment uptake. Third, our data did not distinguish between referrals to facility-based psychosocial counselors, physicians, psychologists, or social workers, and the uptake outcome was restricted to facility-based psychosocial counseling. This meant we were unable to study rates and predictors of patient uptake of specialized care, including prescription of anti-depressants by a PHC physician or receipt of psychotherapy from a psychologist. Further investigation is necessary to assess whether depressive symptom severity and other forms of mental distress predict referral to and uptake of appropriate treatment modalities under a stepped care model (Katon *et al*., 1999). However, given that the introduction or upskilling of a facility-based behavioral counselor is the largest structural change required to adopt the integrated care model, we argue that the rate of facility-based counseling uptake is nevertheless a valuable indicator of the success of integrated care implementation. Finally, this analysis did not assess predictors of patients' subsequent remission from depression. Our focus was on movement through the integrated care model, rather than the effectiveness of mental health treatment. Notably, almost none of the participants in our sample were currently taking anti-depressant medications; this is consistent with other findings from South Africa (Fairall *et al*., 2016).

Nonetheless, our findings have significant implications for the implementation of integrated, PHC-based mental health care in South Africa and other lower-resource health systems implementing similar models, including those in Ethiopia, Zimbabwe, Uganda, and Haiti (Wagner *et al*., 2014; Wissow *et al*., 2014; Chibanda *et al*., 2015; Duffy *et al*., 2017). Though rates of detection and referral closely matched those seen in higher-resource settings, the mental health effects of drop-offs in the depression treatment cascade are likely more egregious in this context. This is because there is a paucity of alternative pathways to effective mental health care in this and similar settings (Seedat *et al*., 2009; Rathod *et al*., 2018), meaning patients with undetected or untreated co-morbid depression will likely remain undetected and untreated. Therefore, targeted implementation strategies are warranted to increase detection and referral rates. For example, brief educational interventions have been shown to improve rates of appropriate nurse detection of, and care for, depressed patients (Bruce *et al*., 2007). Validated screening tools for depression and other CMDs should also be considered for use at patient intake (O'Connor *et al*., 2009); such tools are widely recommended in systems with appropriate systems for subsequent diagnosis, treatment, and follow-up (US Preventive Services Task Force, 2009), and are increasingly available and validated for use in South Africa and other contexts (Bhana *et al*., 2019). Strengthening patient demand for care – for example, by educating and mobilizing communities to improve mental health literacy – will also be critical to improving rates of successful service delivery (Shidhaye *et al*., 2017).

## Conclusion

We assessed the patient-level predictors of movement through the depression care cascade among patients with co-morbid chronic disease and depressive symptoms in Amajuba District, KwaZulu-Natal, South Africa. CNPs detected and referred chronic patients at rates comparable to PHC settings around the world, though substantial gaps persist. CNPs were more likely to detect depressive symptoms in patients in more severe mental distress, suggesting that the CNPs target and reserve care options for patients in greater need. Severity of mental distress was not associated with referral into treatment or counseling uptake, suggesting that higher-level contextual factors may be inhibiting subsequent service delivery in this context. To ensure that integrated mental health care reaches those who need it, strategies for implementation in low-resource settings will need to incorporate targeted efforts to improve rates of detection, referral, and treatment uptake.

## Supplementary material

For supplementary material accompanying this paper visit http://dx.doi.org/10.1017/gmh.2020.11. click here to view supplementary material
