# Supplementary material for: Patient-level predictors of detection of depressive symptoms, referral, and uptake of depression counseling among chronic care patients in KwaZulu-Natal, South Africa
Source: Glob Ment Health (Camb). 2020 Jul 21;7:e18. doi: 10.1017/gmh.2020.11 (PMC7443607; doi:10.1017/gmh.2020.11)
Supplement: Supplementary file 1 [file S2054425120000114sup001.docx]

**Supplemental Table 1: Parsimonious generalized linear mixed effects model estimates of predictors of detection, referral, and uptake**

|  | **Detected** (vs. Not Detected) | | | **Referred** (vs. Not Referred) | | | **Any Uptake** (vs. No Uptake) | | |
| --- | --- | --- | --- | --- | --- | --- | --- | --- | --- |
| *Predictors* | *aOR* | *95% CI* | *p* | *aOR* | *95% CI* | *p* | *aOR* | *95% CI* | *p* |
| (Intercept) | 0.02 | 0.00 – 0.06 | <0.001 | 0.53 | 0.21 – 1.29 | 0.161 | 8.84 | 0.09 – 916.96 | 0.357 |
| Age, years |  |  |  |  |  |  |  |  |  |
| Female |  |  |  |  |  |  |  |  |  |
| Matriculated |  |  |  |  |  |  |  |  |  |
| Employed |  |  |  |  |  |  |  |  |  |
| Income > 2000 ZAR/month |  |  |  |  |  |  |  |  |  |
| Household food insecurity |  |  |  |  |  |  |  |  |  |
| Healthcare use in last 3 months |  |  |  |  |  |  |  |  |  |
| Other PHC Use |  |  |  |  |  |  |  |  |  |
| Hospitalized |  |  |  |  |  |  |  |  |  |
| Prior diagnoses |  |  |  |  |  |  |  |  |  |
| HIV |  |  |  |  |  |  | 4.91 | 0.47 – 51.58 | 0.185 |
| Depression |  |  |  | 2.71 | 0.82 – 8.90 | 0.101 |  |  |  |
| Other chronic disease |  |  |  | 0.44 | 0.23 – 0.84 | 0.013 |  |  |  |
| PACIC score |  |  |  |  |  |  |  |  |  |
| PSS score | 1.1 | 1.05 – 1.15 | <0.001 |  |  |  |  |  |  |
| Oslo score |  |  |  |  |  |  | 0.74 | 0.56 – 0.99 | 0.041 |
| WHODAS score |  |  |  |  |  |  |  |  |  |
| PHQ-9 score | 1.12 | 1.04 – 1.20 | 0.002 |  |  |  | 0.84 | 0.68 – 1.04 | 0.115 |
| Suicidal thoughts | 1.76 | 1.04 – 2.99 | 0.036 |  |  |  |  |  |  |
| AUDIT score | 1.05 | 1.01 – 1.08 | 0.01 |  |  |  |  |  |  |
| **Random Effects** |  |  |  |  |  |  |  |  |  |
| σ^2^ | 3.29 | | | 3.29 | | | 3.29 | | |
| τ_00_ | 1.00 _facility_ | | | 1.39 _facility_ | | | 2.33 _facility_ | | |
| ICC | 0.23 | | | 0.30 | | | 0.41 | | |
| Observations | 409 | | | 207 | | | 75 | | |

Abbreviations

aOR: adjusted odds ratio. CI: confidence interval. ZAR: South African Rand. PHQ-9: Patient Health Questionnaire-9. AUDIT: Alcohol Use Disorders Identification Test. PHC: primary healthcare. PSS: perceived stress scale. WHODAS: WHO Disability Assessment Schedule. ICC: intraclass correlation.
